# When Is a Genuine Multipartite Entanglement Measure Monogamous?

**DOI:** 10.3390/e24030355

**Published:** 2022-02-28

**Authors:** Yu Guo

**Affiliations:** Institute of Quantum Information Science, School of Mathematics and Statistics, Shanxi Datong University, Datong 037009, China; guoyu@sxdtdx.edu.cn

**Keywords:** genuine entanglement, entanglement measure, complete monogamy

## Abstract

A crucial issue in quantum communication tasks is characterizing how quantum resources can be quantified and distributed over many parties. Consequently, entanglement has been explored extensively. However, there are few genuine multipartite entanglement measures and whether it is monogamous is so far unknown. In this work, we explore the complete monogamy of genuine multipartite entanglement measure (GMEM) for which, at first, we investigate a framework for unified/complete GMEM according to the unified/complete multipartite entanglement measure we proposed in 2020. We find a way of inducing unified/complete GMEM from any given unified/complete multipartite entanglement measure. It is shown that any unified GMEM is completely monogamous, and any complete GMEM that is induced by given complete multipartite entanglement measure is completely monogamous. In addition, the previous GMEMs are checked under this framework. It turns out that the genuinely multipartite concurrence is not as good of a candidate as GMEM.

## 1. Introduction

Entanglement is a quintessential manifestation of quantum mechanics and is often considered to be a useful resource for tasks like quantum teleportation or quantum cryptography [1,2,3,4], etc. There has been a tremendous amount of research in the literature aimed at characterizing entanglement in the last three decades [1,2,3,4,5,6,7,8,9]. In an effort to contribute to this line of research, however, the genuine multiparty entanglement, which represents the strongest form of entanglement in many body systems, still remains unexplored or less studied in many facets.

A fundamental issue in this field is to quantify the genuine multipartite entanglement and then analyze the distribution among the different parties. In 2000 [10], Coffman et al. presented a measure of genuine three-qubit entanglement, called “residual tangle”, and discussed the distribution relation for the first time. In 2011, Ma et al. [11] established postulates for a quantity to be a GMEM and gave a genuine measure, called genuinely multipartite concurrence (GMC), by the origin bipartite concurrence. The GMC is further explored in Ref. [12], the generalized geometric measure is introduced in Refs. [13,14], and the average of “residual tangle” and GMC, i.e., $(\tau +C_{gme})/2$ [15], is shown to be genuine multipartite entanglement measures. Another one is the divergence-based genuine multipartite entanglement measure presented in [16,17]. Recently, Ref. [18] introduced a new genuine three-qubit entanglement measure, called *concurrence triangle*, which is quantified as the square root of the area of a triangle deduced by concurrence. Consequently, we improved and supplemented the method in [18] and proposed a general way of defining GMEM in Ref. [19].

The distribution of entanglement is believed to be monogamous, i.e., a quantum system entangled with another system limits its entanglement with the remaining others [20]. There are two methods used in this research. The first one is analyzing monogamy relation based on bipartite entanglement measure, and the second one is based on multipartite entanglement measure. For the former one, considerable efforts have been made in the last two decades [10,21,22,23,24,25,26,27,28,29,30,31,32,33,34,35,36,37,38,39,40]. It is shown that almost all bipartite entanglement measures we know by now are monogamous. In 2020, we established a framework for multipartite entanglement measure and discussed its monogamy relation, which is called complete monogamy relation and tight complete monogamy relation [22]. Under this framework, the distribution of entanglement becomes more clear since it displays a complete hierarchy relation of different subsystems. We also proposed several multipartite entanglement measures and showed that they are completely monogamous.

The situation becomes much more complex when we deal with genuine entanglement, since it associates with not only multiparty system but also the most complex entanglement structure. The main purpose of this work is to establish the framework of unified/complete GMEM, by which we then present the definition of complete monogamy and tight complete monogamy of unified and complete GMEM, respectively. Another aim is to find an approach of deriving GMEM from the multipartite entanglement measure introduced in Ref. [22]. In the next section we list some necessary concepts and the associated notations. In Section 3 we discuss the framework of unified/complete GMEM and give several illustrated examples. Then, in Section 4, we investigate the complete monogamy relation and tight complete monogamy relation for GMEM accordingly. A summary is concluded in the last section.

## 2. Preliminary

For convenience, in this section, we recall the concepts of genuine entanglement, complete multipartite entanglement measure, monogamy relation, complete monogamy relation, and genuine multipartite entanglement measure. In the first subsection, we introduce the coarser relation of multipartite partition by which the following concepts can be easily processed. For simplicity, throughout this paper, we denote by $H^{A_{1}A_{2}\cdots A_{m}}:=H^{A_{1}}⊗H^{A_{2}}⊗\cdots ⊗H^{A_{m}}$ an *m*-partite Hilbert space with finite dimension and by $S^{X}$ we denote the set of density operators acting on $H^{X}$.

### 2.1. Coarser Relation of Multipartite Partition

Let $X_{1}|X_{2}\left|\cdots \right|X_{k}$ be a partition (or called *k*-partition) of $A_{1}A_{2}\cdots A_{m}$, i.e., $X_{s}=A_{s(1)}A_{s(2)}\cdots A_{s(f(s))}$, s(i)<s(j) whenever i<j, and s(p)<t(q) whenever s<t for any possible *p* and *q*, 1≤s,t≤k. For instance, partition AB|C|DE is a 3-partition of ABCDE. Let $X_{1}|X_{2}\left|\cdots \right|X_{k}$ and $Y_{1}|Y_{2}\left|\cdots \right|Y_{l}$ be two partitions of $A_{1}A_{2}\cdots A_{n}$ or subsystem of $A_{1}A_{2}\cdots A_{n}$. $Y_{1}|Y_{2}\left|\cdots \right|Y_{l}$ is said to be *coarser* than $X_{1}|X_{2}\left|\cdots \right|X_{k}$, denoted by

(1) $$\begin{array}{c} X_{1}|X_{2}\left|\cdots \right|X_{k}≻Y_{1}|Y_{2}\left|\cdots \right|Y_{l}, \end{array}$$

if $Y_{1}|Y_{2}\left|\cdots \right|Y_{l}$ can be obtained from $X_{1}|X_{2}\left|\cdots \right|X_{k}$ by one or some of the following ways (the coarser relation was also introduced in Ref. [41], but the the third case in Ref. [41] is a little different from the third item below):

- (C1) Discarding some subsystem(s) of $X_{1}|X_{2}\left|\cdots \right|X_{k}$;
- (C2) Combining some subsystems of $X_{1}|X_{2}\left|\cdots \right|X_{k}$;
- (C3) Discarding some subsystem(s) of some subsystem(s) $X_{k}$ provided that $X_{k}=A_{k(1)}A_{k(2)}\cdots A_{k(f(k))}$ with f(k)≥2.

For example, A|B|C|D|E≻A|B|C|DE≻A|B|C|D≻AB|C|D≻AB|CD, A|B|C|DE≻A|B|DE. Clearly, $X_{1}|X_{2}\left|\cdots \right|X_{k}≻Y_{1}|Y_{2}\left|\cdots \right|Y_{l}$ and $Y_{1}|Y_{2}\left|\cdots \right|Y_{l}≻Z_{1}|Z_{2}\left|\cdots \right|Z_{s}$ imply $X_{1}|X_{2}\left|\cdots \right|X_{k}≻Z_{1}|Z_{2}\left|\cdots \right|Z_{s}$.

Furthermore, if $X_{1}|X_{2}\left|\cdots \right|X_{k}≻Y_{1}|Y_{2}\left|\cdots \right|Y_{l}$, we denote by $\Xi (X_{1}|X_{2}|\cdots |X_{k}-Y_{1}|Y_{2}|\cdots |Y_{l})$ the set of all the partitions that are coarser than $X_{1}|X_{2}\left|\cdots \right|X_{k}$ and either exclude any subsystem of $Y_{1}|Y_{2}\left|\cdots \right|Y_{l}$ or include some but not all subsystems of $Y_{1}|Y_{2}\left|\cdots \right|Y_{l}$. We take the five-partite system ABCDE for example, $\Xi (A|B|CD|E-A|B)=\left\{CD|E,A|CD|E,B|CD|E,A|CD,A|E,B|E,A|C,A|D,B|C,B|D\right\}$.

For more clarity, we fix the following notations. Let $X_{1}|X_{2}\left|\cdots \right|X_{k}$ and $Y_{1}|Y_{2}\left|\cdots \right|Y_{l}$ be partitions of $A_{1}A_{2}\cdots A_{n}$ or subsystem of $A_{1}A_{2}\cdots A_{n}$. We denote by

(2) $$\begin{array}{c} X_{1}|X_{2}\left|\cdots \right|X_{k}{≻}^{a}Y_{1}|Y_{2}\left|\cdots \right|Y_{l} \end{array}$$

for the case of (C1), by

(3) $$\begin{array}{c} X_{1}|X_{2}\left|\cdots \right|X_{k}{≻}^{b}Y_{1}|Y_{2}\left|\cdots \right|Y_{l} \end{array}$$

for the case of of (C2), and in addition by

(4) $$\begin{array}{c} X_{1}|X_{2}\left|\cdots \right|X_{k}{≻}^{c}Y_{1}|Y_{2}\left|\cdots \right|Y_{l} \end{array}$$

for the case of of (C2). For example, $A|B|C|D{≻}^{a}A|B|D{≻}^{a}B|D$, $A|B|C|D{≻}^{b}AC|B|D{≻}^{b}AC|BD$, $A|BC{≻}^{c}A|B$, $A|BC{≻}^{c}A|C$.

### 2.2. Multipartite Entanglement

An *m*-partite pure state $|\psi \rangle \in H^{A_{1}A_{2}\cdots A_{m}}$ is called biseparable if it can be written as $|\psi \rangle ={|\psi \rangle }^{X}⊗{|\psi \rangle }^{Y}$ for some bipartition of $A_{1}A_{2}\cdots A_{m}$. |ψ〉 is said to be *k*-separable if $|\psi \rangle ={|\psi \rangle }^{X_{1}}{|\psi \rangle }^{X_{2}}\cdots {|\psi \rangle }^{X_{k}}$ for some *k*-partition of $A_{1}A_{2}\cdots A_{m}$. |ψ〉 is called fully separable if it is *m*-separable. It is clear that whenever a state is *k*-separable, it is automatically also *l*-separable for all 1<l<k≤m. An *m*-partite mixed state ρ is biseparable if it can be written as a convex combination of biseparable pure states $\rho =\sum_{i}p_{i}|\psi_{i}\rangle \langle \psi_{i}|$, wherein the contained $\left\{|\psi_{i}\rangle \right\}$ can be biseparable with respect to different bipartitions (i.e., a mixed biseparable state does not need to be separable with respect to any particular bipartition). Otherwise it is called genuinely *m*-partite entangled (or called genuinely entangled briefly). We denote by $S_{g}^{A_{1}A_{2}\cdots A_{m}}$ the set of all genuinely entangled states in $S^{A_{1}A_{2}\cdots A_{m}}$. Throughout this paper, for any $\rho \in S^{A_{1}A_{2}\cdots A_{m}}$ and any given *k*-partition $X_{1}|X_{2}\left|\cdots \right|X_{k}$ of $A_{1}A_{2}\cdots A_{m}$, we denote by $\rho^{X_{1}|X_{2}\left|\cdots \right|X_{k}}$ the state for which we consider it as a *k*-partite state with respect to the partition $X_{1}|X_{2}\left|\cdots \right|X_{k}$.

### 2.3. Complete Multipartite Entanglement Measure

A function $E^{\left(m\right)}:S^{A_{1}A_{2}\cdots A_{m}}\to R_{+}$ is called an *m*-partite entanglement measure in literatures [3,42,43] if it satisfies:

- **(E1)**$E^{\left(m\right)}\left(\rho \right)=0$ if ρ is fully separable;
- **(E2)**$E^{\left(m\right)}$ cannot increase under *m*-partite LOCC.

An *m*-partite entanglement measure $E^{\left(m\right)}$ is said to be an *m*-partite entanglement monotone if it is convex and does not increase on average under *m*-partite stochastic LOCC. For simplicity, throughout this paper, if *E* is an entanglement measure (bipartite, or multipartite) for pure states, we define

(5) $$\begin{array}{c} E_{F}\left(\rho \right):=\min\sum_{i}p_{i}E^{\left(m\right)}(|\psi_{i}\rangle ) \end{array}$$

and call it the convex-roof extension of *E*, where the minimum is taken over all pure-state decomposition $\left\{p_{i},|\psi_{i}\rangle \right\}$ of ρ (Sometimes, we use $E^{F}$ to denote $E_{F}$ hereafter). When we take into consideration an *m*-partite entanglement measure, we need discuss whether it is defined uniformly for any *k*-partite system at first, k<m. Let $E^{\left(m\right)}$ be a multipartite entanglement measure (MEM). If $E^{\left(k\right)}$ is uniquely determined by $E^{\left(m\right)}$ for any 2≤k<m, then we call $E^{\left(m\right)}$ a *uniform* MEM. For example, GMC, denoted by $C_{gme}$ [11], is uniquely defined for any *k*, thus it is a uniform GMEM. Recall that,

$$\begin{array}{c} C_{gme}(|\psi \rangle ):=\min_{\gamma_{i}\in \gamma }\sqrt{2\left[1-\mathrm{Tr}{\left(\rho^{A_{\gamma_{i}}}\right)}^{2}\right]} \end{array}$$

for pure state $|\psi \rangle \in H^{A_{1}A_{2}\cdots A_{m}}$, where $\gamma =\{\gamma_{i}\}$ represents the set of all possible bipartitions of $A_{1}A_{2}\cdots A_{m}$, and via the convex-roof extension for mixed states [11]. All the unified MEMs presented in Ref. [22] are uniform MEM. That is, a uniform MEM is series of MEMs that have uniform expressions definitely. A uniform MEM $E^{\left(m\right)}$ is called a *unified* multipartite entanglement measure if it also satisfies the following condition [22]:

- **(E3)***the unification condition*, i.e., $E^{\left(m\right)}$ is consistent with $E^{\left(k\right)}$ for any 2⩽k<m.

The unification condition should be comprehended in the following sense [22]. Let ${|\psi \rangle }^{A_{1}A_{2}\cdots A_{m}}={|\psi \rangle }^{A_{1}A_{2}\cdots A_{k}}{|\psi \rangle }^{A_{k+1}\cdots A_{m}}$, then

$$\begin{array}{c} E^{\left(m\right)}{(|\psi \rangle }^{A_{1}A_{2}\cdots A_{m}})=E^{\left(k\right)}{(|\psi \rangle }^{A_{1}A_{2}\cdots A_{k}})+E^{\left(m-k\right)}{|\psi \rangle }^{A_{k+1}\cdots A_{m}}. \end{array}$$

And

$$\begin{array}{c} E^{\left(m\right)}\left(\rho^{A_{1}A_{2}\cdots A_{m}}\right)=E^{\left(m\right)}\left(\rho^{\pi (A_{1}A_{2}\cdots A_{m})}\right) \end{array}$$

for any $\rho^{A_{1}A_{2}\cdots A_{m}}\in S^{A_{1}A_{2}\cdots A_{m}}$, where π is a permutation of the subsystems. In addition,

$$\begin{array}{c} E^{\left(k\right)}(X_{1}|X_{2}\left|\cdots \right|X_{k})⩾E^{\left(l\right)}(Y_{1}|Y_{2}\left|\cdots \right|Y_{l}) \end{array}$$

for any $\rho^{A_{1}A_{2}\cdots A_{m}}\in S^{A_{1}A_{2}\cdots A_{m}}$ whenever $X_{1}|X_{2}\left|\cdots \right|X_{k}{≻}^{a}Y_{1}|Y_{2}\left|\cdots \right|Y_{l}$, where the vertical bar indicates the split across which the entanglement is measured. A uniform MEM $E^{\left(m\right)}$ is called a *complete* multipartite entanglement measure if it satisfies both **(E3)** above and the following [22]:

- **(E4)**$E^{\left(m\right)}(X_{1}|X_{2}\left|\cdots \right|X_{k})⩾E^{\left(k\right)}(Y_{1}|Y_{2}\left|\cdots \right|Y_{l})$ holds for all $\rho \in S^{A_{1}A_{2}\cdots A_{m}}$ whenever $X_{1}|X_{2}\left|\cdots \right|X_{k}{≻}^{b}Y_{1}|Y_{2}\left|\cdots \right|Y_{l}$.

We need to remark here that, although the partial trace is in fact a special trace-preserving completely positive map, we cannot derive $\rho^{Y_{1}|Y_{2}\left|\cdots \right|Y_{l}}$ from $\rho^{X_{1}|X_{2}\left|\cdots \right|X_{k}}$ by any *k*-partite LOCC for any given $X_{1}|X_{2}\left|\cdots \right|X_{k}≻Y_{1}|Y_{2}\left|\cdots \right|Y_{l}$. Namely, different from that of bipartite case, the unification condition cannot be induced by the *m*-partite LOCC. For any bipartite measure *E*, E(A|BC)≥E(AB) for any $\rho^{ABC}$ since $\rho^{AB}={\mathrm{Tr}}_{C}\rho^{ABC}$ can be obtained by partial trace on part *C* and such a partial trace is in fact a bipartite LOCC acting on A|BC. However, $\rho^{AB}$ cannot be derived from any tripartite LOCC acting on $\rho^{ABC}$. Thus, whether $E^{\left(3\right)}\left(A|BC\right)\geq E^{\left(2\right)}\left(AB\right)$ is unknown.

Several unified tripartite entanglement measures were proposed in Ref. [22]:

$$\begin{array}{ccc} E_{f}^{\left(3\right)}\left(|\psi \rangle \right)\; & = & \;\frac{1}{2}\left[S\left(\rho^{A}\right)+S\left(\rho^{B}\right)+S\left(\rho^{C}\right)\right],\\ \tau^{\left(3\right)}(|\psi \rangle )\; & = & \;3-\mathrm{Tr}{\left(\rho^{A}\right)}^{2}-\mathrm{Tr}{\left(\rho^{B}\right)}^{2}-\mathrm{Tr}{\left(\rho^{C}\right)}^{2},\;\;\;\\ C^{\left(3\right)}(|\psi \rangle )\; & = & \;\sqrt{\tau^{\left(3\right)}(|\psi \rangle )},\\ N^{\left(3\right)}(|\psi \rangle )\; & = & \;{\mathrm{Tr}}^{2}\sqrt{\rho^{A}}+{\mathrm{Tr}}^{2}\sqrt{\rho^{B}}+{\mathrm{Tr}}^{2}\sqrt{\rho^{C}}-3,\\ T_{q}^{\left(3\right)}(|\psi \rangle )\; & = & \;\frac{1}{2}\left[T_{q}\left(\rho^{A}\right)+T_{q}\left(\rho^{B}\right)+T_{q}\left(\rho^{C}\right)\right],\;q>1,\\ R_{\alpha }^{\left(3\right)}(|\psi \rangle )\; & = & \;\frac{1}{2}R_{\alpha }\left(\rho^{A}⊗\rho^{B}⊗\rho^{C}\right),\;0<\alpha <1 \end{array}$$

for pure state $|\psi \rangle \in H^{ABC}$, and then by the convex-roof extension for mixed state $\rho^{ABC}\in S^{ABC}$ (for mixed state, $N^{\left(3\right)}$ is replaced with $N_{F}^{\left(3\right)}$), where $T_{q}\left(\rho \right):={\left(1-q\right)}^{-1}\left[\mathrm{Tr}\left(\rho^{q}\right)-1\right]$ is the Tsallis *q*-entropy, $R_{\alpha }\left(\rho \right):={\left(1-\alpha \right)}^{-1}\ln\left(\mathrm{Tr}\rho^{\alpha }\right)$ is the Rényi α-entropy. In addition [22],

(6) $$N^{\left(3\right)}\left(\rho \right)=∥\rho^{T_{a}}{∥}_{\mathrm{Tr}}+∥\rho^{T_{b}}{∥}_{\mathrm{Tr}}+{∥\rho^{T_{c}}∥}_{\mathrm{Tr}}-3$$

for any $\rho \in S^{ABC}$. $E_{f}^{\left(3\right)}$, $C^{\left(3\right)}$, $\tau^{\left(3\right)}$ and $T_{q}^{\left(3\right)}$ are shown to be complete tripartite entanglement measures while $R_{\alpha }^{\left(3\right)}$, $N^{\left(3\right)}$ and $N_{F}^{\left(3\right)}$ are proved to be unified but not complete tripartite entanglement measures [22].

In Ref. [44], we introduce three unified tripartite entanglement measures (but not complete tripartite entanglement measures) in terms of fidelity:

(7) $$\begin{array}{ccc} E_{F}^{\left(3\right)}\left(|\psi \rangle \right)\; & := & \;1-F\left(|\psi \rangle \langle \psi |,\rho^{A}⊗\rho^{B}⊗\rho^{C}\right),\;\; \end{array}$$

(8) $$\begin{array}{ccc} E_{F^{\prime }}^{\left(3\right)}\left(|\psi \rangle \right)\; & := & \;1-\sqrt{F}\left(|\psi \rangle \langle \psi |,\rho^{A}⊗\rho^{B}⊗\rho^{C}\right),\;\; \end{array}$$

(9) $$\begin{array}{ccc} E_{AF}^{\left(3\right)}\left(|\psi \rangle \right)\; & := & \;1-F_{A}\left(|\psi \rangle \langle \psi |,\rho^{A}⊗\rho^{B}⊗\rho^{C}\right),\;\; \end{array}$$

for any pure state |ψ〉 in $H^{ABC}$, where F is the Uhlmann-Jozsa fidelity F [45,46], which is defined as

(10) $$\begin{array}{c} F\left(\rho ,\sigma \right):={\left(\mathrm{Tr}\sqrt{\sqrt{\rho }\sigma \sqrt{\rho }}\right)}^{2}, \end{array}$$

$\sqrt{F}$ is defined by [47,48,49]

(11) $$\begin{array}{c} \sqrt{F}\left(\rho ,\sigma \right):=\sqrt{F(\rho ,\sigma )}, \end{array}$$

and the *A-fidelity*, $F_{A}$, is the square of the quantum affinity A(ρ,σ) [50,51], i.e.,

(12) $$\begin{array}{c} F_{A}\left(\rho ,\sigma \right):={\left[\mathrm{Tr}\left(\sqrt{\rho }\sqrt{\sigma }\right)\right]}^{2}. \end{array}$$

For mixed states, $E_{F,F}^{\left(3\right)}$, $E_{F^{\prime },F}^{\left(3\right)}$, and $E_{AF,F}^{\left(3\right)}$ are defined by the convex-roof extension as in Equation (5).

### 2.4. Monogamy Relation

For a given bipartite measure *Q* (such as entanglement measure and other quantum correlation measure), *Q* is said to be monogamous (we take the tripartite case for example) if [10,26]

(13) $$\begin{array}{c} Q(A|BC)⩾Q(AB)+Q(AC). \end{array}$$

However, Equation (13) is not valid for many entanglement measures [10,24,52,53] but some power function of *Q* admits the monogamy relation (i.e., $Q^{\alpha }\left(A|BC\right)⩾Q^{\alpha }\left(AB\right)+Q^{\alpha }\left(AC\right)$ for some α>0). In Ref. [23], we address this issue by proposing an improved definition of monogamy (without inequalities) for entanglement measure: A bipartite measure of entanglement *E* is monogamous if for any $\rho \in S^{ABC}$ that satisfies the *disentangling condition*, i.e.,

(14) $$E\left(\rho^{A|BC}\right)=E\left(\rho^{AB}\right),$$

we have that $E(\rho^{AC})=0$, where $\rho^{AB}={\mathrm{Tr}}_{C}\rho^{ABC}$. With respect to this definition, a continuous measure *E* is monogamous according to this definition if and only if there exists 0<α<∞ such that

(15) $$E^{\alpha }\left(\rho^{A|BC}\right)⩾E^{\alpha }\left(\rho^{AB}\right)+E^{\alpha }\left(\rho^{AC}\right)$$

for all ρ acting on the state space $H^{ABC}$ with fixed $\dim H^{ABC}=d<\infty$ (see Theorem 1 in Ref. [23]). Notice that, for these bipartite measures, only the relation between A|BC, AB and AC are revealed, and the global correlation in ABC and the correlation contained in part BC are missed [22]. That is, the monogamy relation in such a sense is not “complete”. For a unified tripartite entanglement measure $E^{\left(3\right)}$, it is said to be *completely monogamous* if for any $\rho \in S^{ABC}$ that satisfies [22]

(16) $$E^{\left(3\right)}\left(\rho^{ABC}\right)=E^{\left(2\right)}\left(\rho^{AB}\right)$$

we have that $E^{\left(2\right)}\left(\rho^{AC}\right)=E^{\left(2\right)}\left(\rho^{BC}\right)=0$. If $E^{\left(3\right)}$ is a continuous unified tripartite entanglement measure. Then, $E^{\left(3\right)}$ is completely monogamous if and only if there exists 0<α<∞ such that [22]

(17) $$\begin{array}{c} E^{\alpha }\left(\rho^{ABC}\right)⩾E^{\alpha }\left(\rho^{AB}\right)+E^{\alpha }\left(\rho^{AC}\right)+E^{\alpha }\left(\rho^{BC}\right) \end{array}$$

for all $\rho^{ABC}\in S^{ABC}$ with fixed $\dim H^{ABC}=d<\infty$, here we omitted the superscript ${}^{\left(2,3\right)}$ of $E^{\left(2,3\right)}$ for brevity. Let $E^{\left(3\right)}$ be a complete MEM. $E^{\left(3\right)}$ is defined to be tightly complete monogamous if for any state $\rho^{ABC}\in S^{ABC}$ that satisfies [22]

(18) $$\begin{array}{c} E^{\left(3\right)}\left(\rho^{ABC}\right)=E^{\left(2\right)}\left(\rho^{A|BC}\right) \end{array}$$

we have $E^{\left(2\right)}\left(\rho^{BC}\right)=0$, which is equivalent to

$$\begin{array}{c} E^{\alpha }\left(\rho^{ABC}\right)⩾E^{\alpha }\left(\rho^{A|BC}\right)+E^{\alpha }\left(\rho^{BC}\right) \end{array}$$

for some α>0. Here we omitted the superscript ${}^{\left(2,3\right)}$ of $E^{\left(2,3\right)}$ for brevity. For the general case of $E^{\left(m\right)}$, one can similarly follow with the same spirit.

### 2.5. Genuine Entanglement Measure

A function $E_{g}^{\left(m\right)}:S^{A_{1}A_{2}\cdots A_{m}}\to R_{+}$ is defined to be a measure of genuine multipartite entanglement if it admits the following conditions [11]:

- **(GE1)**$E_{g}^{\left(m\right)}\left(\rho \right)=0$ for any biseparable $\rho \in S^{A_{1}A_{2}\cdots A_{m}}$;
- **(GE2)**$E_{g}^{\left(m\right)}\left(\rho \right)>0$ for any genuinely entangled state $\rho \in S^{A_{1}A_{2}\cdots A_{m}}$. This item can be weakened as: $E_{g}^{\left(m\right)}\left(\rho \right)⩾0$ for any genuinely entangled state $\rho \in S^{A_{1}A_{2}\cdots A_{m}}$. That is, maybe there exists some state that is genuinely entangled such that $E_{g}^{\left(m\right)}\left(\rho \right)=0$. In such a case, the measure is called not faithful. Otherwise, it is called faithful. For example, the “residual tangle” is not faithful since it is vanished for the *W* state;
- **(GE3)**$E_{g}^{\left(m\right)}\left(\sum_{i}p_{i}\rho_{i}\right)⩽\sum_{i}p_{i}E_{g}^{\left(m\right)}\left(\rho_{i}\right)$ for any $\left\{p_{i},\rho_{i}\right\}$, $\rho_{i}\in S^{A_{1}A_{2}\cdots A_{m}}$, $p_{i}>0$, $\sum_{i}p_{i}=1$;
- **(GE4)**$E_{g}^{\left(m\right)}\left(\rho \right)⩾E_{g}^{\left(m\right)}\left(\rho^{\prime }\right)$ for any *m*-partite LOCC ε, $\varepsilon \left(\rho \right)=\rho^{\prime }$.

Note that **(GE4)** implies that $E_{g}^{\left(m\right)}$ is invariant under local unitary transformations. $E_{g}^{\left(m\right)}$ is said to be a genuine multipartite entanglement monotone if it does not increase on average under *m*-partite stochastic LOCC. For example, $C_{gme}$ is a GMEM.

## 3. Complete Genuine Multipartite Entanglement Measure

Analogous to that of unified/complete multipartite entanglement measure established in Ref. [22], we discuss the unification condition and the hierarchy condition for genuine multipartite entanglement measure in this section. We start out with an observation of the examples. Let |ψ〉 be an *m*-partite pure state in $H^{A_{1}A_{2}\cdots A_{m}}$. Recall that, the multipartite entanglement of formation $E_{f}^{\left(m\right)}$ is defined as [22]

$$\begin{array}{ccc} E_{f}^{\left(m\right)}(|\psi \rangle )\; & := & \;\frac{1}{2}\sum_{i=1}^{m}S\left(\rho_{A_{i}}\right), \end{array}$$

where $\rho_{X}:={\mathrm{Tr}}_{\bar{X}}(|\psi \rangle \langle \psi |)$. We define

(19) $$\begin{array}{ccc} E_{g-f}^{\left(m\right)}(|\psi \rangle )\; & := & \;\frac{1}{2}\delta (|\psi \rangle )\sum_{i=1}^{m}S\left(\rho_{A_{i}}\right), \end{array}$$

where δ(ρ)=0 if ρ is biseparable up to some bi-partition and δ(ρ)=1 if ρ is not biseparable up to any bi-partition. For mixed state, it is defined by the convex-roof extension. Obviously, $E_{g-f}^{\left(m\right)}$ is a uniform GMEM since $I(A_{1}:A_{2}:\cdots :A_{n})⩾0$ for any *n* [54], where $I\left(A_{1}:A_{2}:\cdots :A_{n}\right):=\sum_{k=1}^{n}S\left(\rho_{A_{k}}\right)-S\left(A_{1}A_{2}\cdots A_{n}\right)=S\left(\rho^{A_{1}A_{2}\cdots A_{n}}∥\rho^{A_{1}}⊗\rho^{A_{2}}⊗\cdots \rho^{A_{n}}\right)⩾0$. The following properties are straightforward: For any $\rho^{A_{1}A_{2}\cdots A_{m}}\in S_{g}^{A_{1}A_{2}\cdots A_{m}}$,

$$\begin{array}{c} E_{g-f}^{\left(k\right)}(X_{1}|X_{2}\left|\cdots \right|X_{k})>E_{g-f}^{\left(l\right)}(Y_{1}|Y_{2}\left|\cdots \right|Y_{l}) \end{array}$$

for any $X_{1}|X_{2}\left|\cdots \right|X_{k}{≻}^{b}Y_{1}|Y_{2}\left|\cdots \right|Y_{l}$. It is worth noting that, for any uniform GMEM $E_{g}^{\left(m\right)}$, we cannot require $E_{g}^{\left(k\right)}(X_{1}|X_{2}\left|\cdots \right|X_{k})=E_{g}^{\left(l\right)}(Y_{1}|Y_{2}\left|\cdots \right|Y_{l})$ for any $\rho \in S_{g}^{A_{1}A_{2}\cdots A_{m}}$ and any $X_{1}|X_{2}\left|\cdots \right|X_{k}{≻}^{a}Y_{1}|Y_{2}\left|\cdots \right|Y_{l}$. For example, if $E_{g}^{\left(4\right)}\left(\rho^{ABCD}\right)=E_{g}^{\left(3\right)}\left(\rho^{ABC}\right)$ for some $\rho^{ABCD}\in S_{g}^{ABCD}$, then the entanglement between part ABC and part *D* is zero, which means that $\rho^{ABCD}$ is biseparable with respect to the partition ABC|D—a contradiction. In addition, let ${|\psi \rangle }^{ABC}$ be a tripartite genuine entangled state in $H^{ABC}$, then ${|\psi \rangle }^{ABC}{|\psi \rangle }^{D}$ is not a four-partite genuine entangled state, i.e.,

$$\begin{array}{c} E_{g}^{\left(4\right)}{(|\psi \rangle }^{ABC}{|\psi \rangle }^{D})=0, \end{array}$$

but $E_{g}^{\left(3\right)}{(\psi \rangle }^{ABC})>0$ provided that $E_{g}^{\left(3\right)}$ is faithful. That is, the genuine multipartite entanglement measure is not necessarily decreasing under the discarding of the subsystem. However, for the genuine entangled state, it is decreasing definitely. From these observations, we give the following definition.

**Definition** **1.** *Let $E_{g}^{\left(m\right)}$ be a uniform genuine entanglement measure. If it satisfies the unification condition, i.e.,*

(20) $$\begin{array}{c} E_{g}^{\left(m\right)}\left(A_{1}A_{2}\cdots A_{m}\right)=E_{g}^{\left(m\right)}\left(\pi (A_{1}A_{2}\cdots A_{m})\right) \end{array}$$

*and*

(21) $$\begin{array}{c} E_{g}^{\left(k\right)}(X_{1}|X_{2}\left|\cdots \right|X_{k})>E_{g}^{\left(l\right)}(Y_{1}|Y_{2}\left|\cdots \right|Y_{l}) \end{array}$$

*for any $\rho \in S_{g}^{A_{1}A_{2}\cdots A_{m}}$ whenever $X_{1}|X_{2}\left|\cdots \right|X_{k}{≻}^{a}Y_{1}|Y_{2}\left|\cdots \right|Y_{l}$, we call $E_{g}^{\left(m\right)}$ a unified genuine multipartite entanglement measure, where π(·) denotes the permutation of the subsystems.*

For any $\rho \in S_{g}^{A_{1}A_{2}\cdots A_{m}}$, if $X_{1}|X_{2}\left|\cdots \right|X_{k}{≻}^{b}Y_{1}|Y_{2}\left|\cdots \right|Y_{l}$, We expect any unified GMEM satisfies $E_{g}^{\left(k\right)}(X_{1}|X_{2}\left|\cdots \right|X_{k})⩾E_{g}^{\left(l\right)}(Y_{1}|Y_{2}\left|\cdots \right|Y_{l})$ since ‘some amount of entanglement’ may be hided in the combined subsystem. For example, the quantity $E_{g}^{\left(3\right)}\left(AB|C|D\right)$ cannot report the entanglement contained between subsystems *A* and *B*. We thus present the following definition.

**Definition** **2.** *Let $E_{g}^{\left(m\right)}$ be a unified GMEM. If $E_{g}^{\left(m\right)}$ admits the hierarchy condition, i.e.,*

(22) $$\begin{array}{c} E_{g}^{\left(k\right)}(X_{1}|X_{2}\left|\cdots \right|X_{k})⩾E_{g}^{\left(l\right)}(Y_{1}|Y_{2}\left|\cdots \right|Y_{l}) \end{array}$$

*for any $\rho \in S_{g}^{A_{1}A_{2}\cdots A_{m}}$ whenever $X_{1}|X_{2}\left|\cdots \right|X_{k}{≻}^{b}Y_{1}|Y_{2}\left|\cdots \right|Y_{l}$, then it is said to be a complete genuine multipartite entanglement measure.*

We remark here that, for any given uniform GMEM $E_{g}^{\left(m\right)}$,

(23) $$\begin{array}{c} E_{g}^{\left(k\right)}(X_{1}|X_{2}\left|\cdots \right|X_{k})⩾E_{g}^{\left(k\right)}(X_{1}^{\prime }|X_{2}^{\prime }\left|\cdots \right|X_{k}^{\prime }) \end{array}$$

holds for any $\rho \in S_{g}^{A_{1}A_{2}\cdots A_{m}}$ whenever $X_{1}|X_{2}\left|\cdots \right|X_{k}{≻}^{c}X_{1}^{\prime }|X_{2}^{\prime }\left|\cdots \right|X_{k}^{\prime }$ since $\rho^{X_{1}^{\prime }|X_{2}^{\prime }\left|\cdots \right|X_{k}^{\prime }}$ is obtained from $\rho^{X_{1}|X_{2}\left|\cdots \right|X_{k}}$ by partial trace and such a partial trace is indeed a *k*-partite LOCC, 2≤k≤m. That is, a complete GMEM is a series of GMEMs that are compatible in the following sense: Not only the genuine entanglement contained in the global system and that of any subsystem or new partition of the global system are comparable but also the genuine entanglement in any subsystems with the coarser relation can be compared with each other. Of course, the genuine entanglement should be decreasing whenever the system is coarsening, as one may expect. By definition, $E_{g-f}^{\left(m\right)}$ is a complete GMEM. We just take $E_{g-f}^{\left(m\right)}$ for example. For the three-qubit GHZ state $|GHZ\rangle =\frac{1}{\sqrt{2}}(|000\rangle +\left|111\rangle \right)$,

$$\begin{array}{c} E_{g-f}^{\left(3\right)}(|GHZ\rangle )=\frac{3}{2}>E_{g-f}^{\left(2\right)}{(|GHZ\rangle }^{A|BC})=1>E_{g-f}^{\left(2\right)}\left(\rho^{AB}\right)=0, \end{array}$$

and for the W state $|W\rangle =\frac{1}{\sqrt{3}}(|100\rangle +|010\rangle +\left|001\rangle \right)$, it is straightforward that

$$\begin{array}{c} E_{g-f}^{\left(3\right)}(|W\rangle )=\frac{3}{2}\log_{2}3-1>E_{g-f}^{\left(2\right)}{(|W\rangle }^{A|BC})=\log_{2}3-\frac{2}{3}>E_{g-f}^{\left(2\right)}\left(\rho^{AB}\right)=\frac{2}{3}. \end{array}$$

In general, the equality in Equation (23) does not hold, i.e., the genuine entanglement decreases strictly under coarser relation (C3). For example, if $E{(|\psi \rangle }^{A|BC})=E\left(\rho^{AB}\right)$, then ${|\psi \rangle }^{ABC}$ is biseparable for almost all bipartite entanglement measures *E* so far [36].

It is clear that $C_{gme}$ is not a complete GMEM since it does not satisfy the hierarchy condition (22). We take a four-partite state for example. Let

$$\begin{array}{c} |\psi \rangle =\frac{\sqrt{5}}{4}|0000\rangle +\frac{1}{4}|1111\rangle +\frac{\sqrt{5}}{4}|0100\rangle +\frac{\sqrt{5}}{4}|1010\rangle , \end{array}$$

then $C_{gme}{(|\psi \rangle )=C(|\psi \rangle }^{ABC|D})=\frac{\sqrt{15}}{8}<C{(|\psi \rangle }^{AB|CD})=\frac{\sqrt{65}}{8}$. In general, $C_{gme}$ is not even a unified GMEM since we can not guarantee that unification condition (21) holds true.

We now turn to find unified/complete GMEM. $E_{g-f}^{\left(m\right)}$ is derived from unified/complete multipartite entanglement measures $E_{f}^{\left(m\right)}$. This motivates us to obtain unified/complete GMEMs from the unified/complete MEMs.

**Proposition** **1.** *Let $E^{\left(m\right)}$ be a unified/complete multipartite entanglement measure (resp. monotone), and define*

(24) $$\begin{array}{c} E_{g-F}^{\left(m\right)}\left(\rho \right):=\min_{\left\{p_{i},|\psi_{i}\rangle \right\}}\sum p_{i}\delta (|\psi_{i}\rangle )E^{\left(m\right)}(|\psi_{i}\rangle ) \end{array}$$

*whenever $E_{F}^{\left(m\right)}=\min_{\left\{p_{i},|\psi_{i}\rangle \right\}}\sum p_{i}E^{\left(m\right)}(|\psi_{i}\rangle )$ and*

(25) $$\begin{array}{c} E_{g}^{\left(m\right)}\left(\rho \right):=\delta \left(\rho \right)E^{\left(m\right)}\left(\rho \right) \end{array}$$

*whenever $E^{\left(m\right)}$ is not defined by the convex-roof extension for mixed state, where the minimum is taken over all pure-state decomposition $\left\{p_{i},|\psi_{i}\rangle \right\}$ of $\rho \in S^{A_{1}A_{2}\cdots A_{m}}$, δ(ρ)=1 whenever ρ is genuinely entangled and δ(ρ)=0 otherwise. Then, $E_{g}^{\left(m\right)}$ is a unified/complete genuine multipartite entanglement measure (resp. monotone).*

**Proof.**  It is clear that $E_{g-F}^{\left(m\right)}$ and $E_{g}^{\left(m\right)}$ satisfy the unification condition (resp. hierarchy condition) on $S_{g}^{A_{1}A_{2}\cdots A_{m}}$ whenever $E^{\left(m\right)}$ satisfies the unification condition (resp. hierarchy condition) on $S^{A_{1}A_{2}\cdots A_{m}}$. □

Consequently, according to Proposition 1, we get

$$\begin{array}{ccc} \tau_{g}^{\left(3\right)}(|\psi \rangle )\; & = & \;\delta (|\psi \rangle )\left[3-\mathrm{Tr}{\left(\rho^{A}\right)}^{2}-\mathrm{Tr}{\left(\rho^{B}\right)}^{2}-\mathrm{Tr}{\left(\rho^{C}\right)}^{2}\right],\;\;\;\\ C_{g}^{\left(3\right)}(|\psi \rangle )\; & = & \;\sqrt{\tau_{g}^{\left(3\right)}(|\psi \rangle )},\\ N_{g}^{\left(3\right)}(|\psi \rangle )\; & = & \;\delta (|\psi \rangle )\left[{\mathrm{Tr}}^{2}\sqrt{\rho^{A}}+{\mathrm{Tr}}^{2}\sqrt{\rho^{B}}+{\mathrm{Tr}}^{2}\sqrt{\rho^{C}}-3\right],\\ T_{g-q}^{\left(3\right)}(|\psi \rangle )\; & = & \;\frac{1}{2}\delta (|\psi \rangle )\left[T_{q}\left(\rho^{A}\right)+T_{q}\left(\rho^{B}\right)+T_{q}\left(\rho^{C}\right)\right],\;q>1,\\ R_{g-\alpha }^{\left(3\right)}(|\psi \rangle )\; & = & \;\frac{1}{2}\delta (|\psi \rangle )R_{\alpha }\left(\rho^{A}⊗\rho^{B}⊗\rho^{C}\right),\;0<\alpha <1,\\ E_{g-F}^{\left(3\right)}\left(|\psi \rangle \right)\; & = & \;\delta (|\psi \rangle )\left[1-F\left(|\psi \rangle \langle \psi |,\rho^{A}⊗\rho^{B}⊗\rho^{C}\right)\right],\\ E_{g-F^{\prime }}^{\left(3\right)}\left(|\psi \rangle \right)\; & = & \;\delta (|\psi \rangle )\left[1-\sqrt{F}\left(|\psi \rangle \langle \psi |,\rho^{A}⊗\rho^{B}⊗\rho^{C}\right)\right],\\ E_{g-AF}^{\left(3\right)}\left(|\psi \rangle \right)\; & = & \;\delta (|\psi \rangle )\left[1-F_{A}\left(|\psi \rangle \langle \psi |,\rho^{A}⊗\rho^{B}⊗\rho^{C}\right)\right], \end{array}$$

for pure states, and define by the convex-roof extension for the mixed states (for mixed state, where $N_{g}^{\left(3\right)}$ is replaced with the convex-roof extension of $N_{g}^{\left(3\right)}$, $N_{g-F}^{\left(3\right)}$), and

$$\begin{array}{c} N_{g}^{\left(3\right)}\left(\rho \right)=\delta \left(\rho \right)\left(∥\rho^{T_{a}}{∥}_{\mathrm{Tr}}+∥\rho^{T_{b}}{∥}_{\mathrm{Tr}}+{∥\rho^{T_{c}}∥}_{\mathrm{Tr}}-3\right) \end{array}$$

for any $\rho \in S^{ABC}$. These tripartite measures, except for $N_{g}^{\left(3\right)}$ are in fact special cases of $E_{g-123}^{F}$ in Ref. [19]. Generally, we can define

$$\begin{array}{ccc} \tau_{g}^{\left(m\right)}(|\psi \rangle )\; & = & \;\delta (|\psi \rangle )\left[m-\sum_{i}\mathrm{Tr}{\left(\rho^{A_{i}}\right)}^{2}\right],\;\;\;\\ C_{g}^{\left(m\right)}(|\psi \rangle )\; & = & \;\sqrt{\tau_{g}^{\left(m\right)}(|\psi \rangle )},\\ N_{g}^{\left(m\right)}(|\psi \rangle )\; & = & \;\delta (|\psi \rangle )\left[\sum_{i}{\mathrm{Tr}}^{2}\sqrt{\rho^{A_{i}}}-m\right],\\ T_{g-q}^{\left(m\right)}(|\psi \rangle )\; & = & \;\frac{1}{2}\delta (|\psi \rangle )\sum_{i}T_{q}\left(\rho^{A_{i}}\right),\;q>1,\\ R_{g-\alpha }^{\left(m\right)}(|\psi \rangle )\; & = & \;\frac{1}{2}\delta (|\psi \rangle )R_{\alpha }\left(\underset{i}{⨂}\rho^{A_{i}}\right),\;0<\alpha <1,\\ E_{g-F}^{\left(m\right)}\left(|\psi \rangle \right)\; & = & \;\delta (|\psi \rangle )\left[1-F\left(|\psi \rangle \langle \psi |,\underset{i}{⨂}\rho^{A_{i}}\right)\right],\\ E_{g-F^{\prime }}^{\left(m\right)}\left(|\psi \rangle \right)\; & = & \;\delta (|\psi \rangle )\left[1-\sqrt{F}\left(|\psi \rangle \langle \psi |,\underset{i}{⨂}\rho^{A_{i}}\right)\right],\\ E_{g-AF}^{\left(m\right)}\left(|\psi \rangle \right)\; & = & \;\delta (|\psi \rangle )\left[1-F_{A}\left(|\psi \rangle \langle \psi |,\underset{i}{⨂}\rho^{A_{i}}\right)\right], \end{array}$$

for pure states and define by the convex-roof extension for the mixed states (for mixed state, $N_{g}^{\left(m\right)}$ is replaced with $N_{g-F}^{\left(m\right)}$), and

$$\begin{array}{c} N_{g}^{\left(m\right)}\left(\rho \right)=\delta \left(\rho \right)\left({∥\sum_{i}\rho^{T_{i}}∥}_{\mathrm{Tr}}-m\right) \end{array}$$

for any $\rho \in S^{A_{1}A_{2}\cdots A_{m}}$. According to Proposition 1, together with Theorem 5 in Ref. [22], the statement below is straightforward.

**Proposition** **2.** *$E_{g-f}^{\left(m\right)}$, $\tau_{g}^{\left(m\right)}$, $C_{g}^{\left(m\right)}$, and $T_{g-q}^{\left(m\right)}$ are complete genuine multipartite entanglement monotones while $R_{g-\alpha }^{\left(m\right)}$, $N_{g-F}^{\left(m\right)}$, $N_{g}^{\left(m\right)}$, $E_{g-F}^{\left(m\right)}$, $E_{g-F^{\prime }}^{\left(m\right)}$, and $E_{g-AF}^{\left(m\right)}$ are unified genuine multipartite entanglement monotones, but not complete genuine multipartite entanglement monotones.*

Very recently, we proposed the following genuine four-partite entanglement measures [19]. Let *E* be a bipartite entanglement measure and let

(26) $$\begin{array}{c} E_{g-1234(2)}(|\psi \rangle ):=\delta (|\psi \rangle )\sum_{i}x_{i}^{\left(2\right)} \end{array}$$

for any given $|\psi \rangle \in H^{ABCD}$, where $E{(|\psi \rangle }^{AB|CD})=x_{1}^{\left(2\right)}$, $E{(|\psi \rangle }^{A|BCD})=x_{2}^{\left(2\right)}$, $E{(|\psi \rangle }^{AC|BD})=x_{3}^{\left(2\right)}$, $E{(|\psi \rangle }^{ABC|D})=x_{4}^{\left(2\right)}$, $E{(|\psi \rangle }^{AD|BC})=x_{5}^{\left(2\right)}$, $E{(|\psi \rangle }^{B|ACD})=x_{6}^{\left(2\right)}$, $E{(|\psi \rangle }^{C|ABD})=x_{7}^{\left(2\right)}$. Then $E_{g-1234(2)}^{F}$ is a genuine four-partite entanglement measure. Let $E^{\left(3\right)}$ be a tripartite entanglement measure,

(27) $$\begin{array}{c} E_{g-1234(3)}(|\psi \rangle )=\delta (|\psi \rangle )\sum_{i}x_{i}^{\left(3\right)} \end{array}$$

for any given $|\psi \rangle \in S^{ABCD}$, where $E^{\left(3\right)}\left(\rho^{A|B|CD}\right)=x_{1}^{\left(3\right)}$, $E^{\left(3\right)}\left(\rho^{A|BC|D}\right)=x_{2}^{\left(3\right)}$, $E^{\left(3\right)}\left(\rho^{AC|B|D}\right)=x_{3}^{\left(3\right)}$, $E^{\left(3\right)}\left(\rho^{AB|C|D}\right)=x_{4}^{\left(3\right)}$, $E^{\left(3\right)}\left(\rho^{AD|B|C}\right)=x_{5}^{\left(3\right)}$, $E^{\left(3\right)}\left(\rho^{A|BD|C}\right)=x_{6}^{\left(3\right)}$. It is clear that $E_{g-1234(3)}^{F}$ is a genuine four-partite entanglement measure but not uniform GMEM.

Generally, we can define $E_{g-1234\cdots m(2)}^{F}$ by the same way, and it is a uniform GMEM. We check below that $E_{g-1234\cdots m(2)}^{F}$ is a complete GMEM whenever *E* is an entanglement monotone. We only need to discuss the case of m=4, and the general cases can be argued similarly. For any genuine entangled pure state $|\psi \rangle \in H^{ABCD}$, and any bipartite entanglement monotone *E*, it is clear that $E_{g-1234(2)}(|\psi \rangle )>E^{F}\left(\rho^{XY}\right)$ for any {X,Y}∈{A,B,C,D}. For any pure state decomposition of $\rho^{ABC}$, $\rho^{ABC}=\sum_{i}p_{i}|\psi_{i}\rangle \langle \psi_{i}|$, we have $E{(|\psi \rangle }^{A|BCD})⩾\sum_{i}p_{i}E{(|\psi_{i}\rangle }^{A|BC})$, $E{(|\psi \rangle }^{AB|CD})⩾\sum_{i}p_{i}E{(|\psi_{i}\rangle }^{AB|C})$, and $E{(|\psi \rangle }^{B|ACD})⩾\sum_{i}p_{i}E{(|\psi_{i}\rangle }^{B|AC})$ since any ensemble $\left\{p_{i},|\psi_{i}\rangle \right\}$ can be derived by LOCC from |ψ〉. It follows that $E_{g-1234(2)}(|\psi \rangle )>E_{g-123(2)}^{F}\left(\rho^{ABC}\right)$. By symmetry of the subsystems, we get that the unification condition is valid for pure state. For mixed state $\rho \in S_{g}^{ABCD}$, we let

$$\begin{array}{c} E_{g-1234(2)}^{F}\left(\rho \right)=\sum_{j}p_{j}E_{g-1234(2)}(|\phi_{j}\rangle ) \end{array}$$

for some decomposition $\rho =\sum_{j}p_{j}|\phi_{j}\rangle \langle \phi_{j}|$. Then

$$\begin{array}{c} E_{g-1234(2)}(|\phi_{j}\rangle )⩾E_{g-123(2)}^{F}\left(\rho_{j}^{ABC}\right) \end{array}$$

for any *j*, where $\rho_{j}^{ABC}={\mathrm{Tr}}_{D}(|\phi_{j}\rangle \langle \phi_{j}|)$. Therefore

$$\begin{array}{c} E_{g-1234(2)}^{F}\left(\rho \right)=\sum_{j}p_{j}E_{g-1234(2)}(|\phi_{j}\rangle )⩾\sum_{j}p_{j}E_{g-123(2)}^{F}\left(\rho_{j}^{ABC}\right)⩾E_{g-123(2)}^{F}\left(\rho^{ABC}\right) \end{array}$$

as desired. In addition, it is clear that

(28) $$\begin{array}{c} E_{g-123(2)}^{F}\left(\rho^{ABC}\right)>E^{F}\left(\rho^{AB}\right) \end{array}$$

for any $\rho \in S_{g}^{ABCD}$. That is, $E_{g-1234\cdots m(2)}^{F}$ is a unified GMEM. The hierarchy condition is obvious. Thus, $E_{g-1234\cdots m(2)}^{F}$ is a complete GMEM whenever *E* is an entanglement monotone.

**Remark** **1.** *It is clear that, for $E_{g-1234\cdots m(2)}^{F}$, the inequality in Equation (22) is a strict inequality, i.e.,*

(29) $$\begin{array}{c} E_{g}^{\left(k\right)}(X_{1}|X_{2}\left|\cdots \right|X_{k})>E_{g}^{\left(l\right)}(Y_{1}|Y_{2}\left|\cdots \right|Y_{l}) \end{array}$$

*for any $\rho \in S_{g}^{A_{1}A_{2}\cdots A_{m}}$ whenever $X_{1}|X_{2}\left|\cdots \right|X_{k}{≻}^{b}Y_{1}|Y_{2}\left|\cdots \right|Y_{l}$. In addition, according to the proof of Proposition 4 in Ref. [22], Equation (22) holds for $E_{g-f}^{\left(m\right)}$, $\tau_{g}^{\left(m\right)}$, $C_{g}^{\left(m\right)}$, and $T_{g-q}^{\left(m\right)}$. Namely, in general, there does not exist $\rho \in S_{g}^{A_{1}A_{2}\cdots A_{m}}$ such that $E_{g}^{\left(k\right)}(X_{1}|X_{2}\left|\cdots \right|X_{k})=E_{g}^{\left(l\right)}(Y_{1}|Y_{2}\left|\cdots \right|Y_{l})$ holds, $X_{1}|X_{2}\left|\cdots \right|X_{k}{≻}^{b}Y_{1}|Y_{2}\left|\cdots \right|Y_{l}$.*

## 4. Complete Monogamy of Genuine Multipartite Entanglement Measure

We are now ready to discuss the complete monogamy relation of GMEM. By the previous arguments, the genuine multipartite entanglement does not necessarily decrease when discarding the subsystem. However, for the genuine entangled state, it does decrease. We thus conclude the following definition of complete monogamy for genuine entanglement measure.

**Definition** **3.** *Let $E_{g}^{\left(m\right)}$ be a uniform GMEM. We call $E_{g}^{\left(m\right)}$ completely monogamous if for any $\rho \in S_{g}^{A_{1}A_{2}\cdots A_{m}}$ we have*

(30) $$\begin{array}{c} E_{g}^{\left(k\right)}\left(\rho^{X_{1}|X_{2}\left|\cdots \right|X_{k}}\right)>E_{g}^{\left(l\right)}\left(\rho^{Y_{1}|Y_{2}\left|\cdots \right|Y_{l}}\right) \end{array}$$

*holds for all $X_{1}|X_{2}\left|\cdots \right|X_{k}{≻}^{a}Y_{1}|Y_{2}\left|\cdots \right|Y_{l}$.*

That is, any unified GMEM is completely monogamous. Moreover, according to the proof of Theorem 1 in Ref. [23], we can get the equivalent statement of complete monogamy for continuous genuine tripartite entanglement measure (the general *m*-partite case can be followed in the same way).

**Proposition** **3.** *Let $E_{g}^{\left(3\right)}$ be a continuous uniform genuine tripartite entanglement measure. Then, $E_{g}^{\left(3\right)}$ is completely monogamous if and only if there exists 0<α<∞ such that*

(31) $$\begin{array}{c} E_{g}^{\alpha }\left(\rho^{ABC}\right)>E^{\alpha }\left(\rho^{AB}\right)+E^{\alpha }\left(\rho^{AC}\right)+E^{\alpha }\left(\rho^{BC}\right) \end{array}$$

*for all $\rho^{ABC}\in S_{g}^{ABC}$ with fixed $\dim H^{ABC}=d<\infty$, here we omitted the superscript ${}^{\left(3\right)}$ of $E^{\left(3\right)}$ for brevity.*

Analogously, for the four-partite case, if $E_{g}^{\left(4\right)}$ is a continuous uniform GMEM, then $E_{g}^{\left(4\right)}$ is completely monogamous if and only if there exist 0<α,β<∞ such that

(32) $$\begin{array}{ccc} E_{g}^{\alpha }\left(\rho^{ABCD}\right)\; & > & \;E_{g}^{\alpha }\left(\rho^{ABC}\right)+E_{g}^{\alpha }\left(\rho^{ABD}\right)+E_{g}^{\alpha }\left(\rho^{ACD}\right)+E_{g}^{\alpha }\left(\rho^{BCD}\right), \end{array}$$

(33) $$\begin{array}{ccc} E_{g}^{\beta }\left(\rho^{ABCD}\right)\; & > & \;E^{\beta }\left(\rho^{AB}\right)+E^{\beta }\left(\rho^{BC}\right)+E^{\beta }\left(\rho^{AC}\right)+E^{\beta }\left(\rho^{BD}\right)+E^{\beta }\left(\rho^{AD}\right)+E^{\beta }\left(\rho^{CD}\right)\;\;\;\; \end{array}$$

for all $\rho^{ABCD}\in S_{g}^{ABCD}$ with fixed $\dim H^{ABC}=d<\infty$, here we omitted the superscript ${}^{\left(3,4\right)}$ of $E^{\left(3,4\right)}$ for brevity. Since $C_{gme}$ may not be a unified GMEM, we conjecture that $C_{gme}$ is not completely monogamous.

As a counterpart to the tightly complete monogamous relation of the complete multipartite entanglement measure in Ref. [22], we give the following definition.

**Definition** **4.** *Let $E_{g}^{\left(m\right)}$ be a complete GMEM. We call $E_{g}^{\left(m\right)}$ tightly complete monogamous if it satisfies the genuine disentangling condition, i.e., either for any $\rho \in S_{g}^{A_{1}A_{2}\cdots A_{m}}$ that satisfies*

(34) $$\begin{array}{c} E_{g}^{\left(k\right)}\left(X_{1}|X_{2}\left|\cdots \right|X_{k}\right)=E_{g}^{\left(l\right)}\left(Y_{1}|Y_{2}\left|\cdots \right|Y_{l}\right) \end{array}$$

*we have that*

(35) $$\begin{array}{c} E_{g}^{\left(*\right)}\left(\Gamma \right)=0 \end{array}$$

*holds for all $\Gamma \in \Xi (X_{1}|X_{2}|\cdots |X_{k}-Y_{1}|Y_{2}|\cdots |Y_{l})$, or*

(36) $$\begin{array}{c} E_{g}^{\left(k\right)}\left(X_{1}|X_{2}\left|\cdots \right|X_{k}\right)>E_{g}^{\left(l\right)}\left(Y_{1}|Y_{2}\left|\cdots \right|Y_{l}\right) \end{array}$$

holds for any $\rho \in S_{g}^{A_{1}A_{2}\cdots A_{m}}$, where $X_{1}|X_{2}\left|\cdots \right|X_{k}{≻}^{b}Y_{1}|Y_{2}\left|\cdots \right|Y_{l}$, and the superscript (*) is associated with the partition Γ, e.g., if Γ is a n-partite partition, then (*)=(n).

Definitions 3 and 4 mean that, if $E_{g}^{\left(k\right)}\left(X_{1}|X_{2}\left|\cdots \right|X_{k}\right)\approx E_{g}^{\left(l\right)}\left(Y_{1}|Y_{2}\left|\cdots \right|Y_{l}\right)$, then $E_{g}^{\left(*\right)}\left(\Gamma \right)\approx 0$ for any $\Gamma \in \Xi (X_{1}|X_{2}|\cdots |X_{k}-Y_{1}|Y_{2}|\cdots |Y_{l})$. This fact can make ensure the security of quantum communication tasks, which rely on genuine entanglement as the resource: Whenever $E_{g}^{\left(k\right)}\left(X_{1}|X_{2}\left|\cdots \right|X_{k}\right)\approx E_{g}^{\left(l\right)}\left(Y_{1}|Y_{2}\left|\cdots \right|Y_{l}\right)$, the joint information in subsystems $\Gamma \in \Xi (X_{1}|X_{2}|\cdots |X_{k}-Y_{1}|Y_{2}|\cdots |Y_{l})$ is nearly zero, i.e., we could choose such an entangled state when we would like to prevent subsystem Γ in sharing the information based on the genuine entanglement or from any evegetting information from subsystem Γ.

**Remark** **2.** *According to Remark 1, for $E_{g-f}^{\left(m\right)}$, $\tau_{g}^{\left(m\right)}$, $C_{g}^{\left(m\right)}$, $T_{g-q}^{\left(m\right)}$, and $E_{g-1234\cdots m(2)}^{F}$, the case of Equation (34) cannot occur, so they are tightly complete monogamous. We conjecture that the case of Equation (34) cannot occur for any complete GMEM. In such a sense, any complete GMEM is tightly complete monogamous.*

For example, if $E_{g}^{\left(3\right)}$ is a complete GMEM, then $E_{g}^{\left(3\right)}$ is tightly complete monogamous if for any $\rho^{ABC}\in S_{g}^{ABC}$ that satisfies

(37) $$\begin{array}{c} E_{g}^{\left(3\right)}\left(\rho^{ABC}\right)=E^{\left(2\right)}\left(\rho^{A|BC}\right) \end{array}$$

we have $E^{\left(2\right)}\left(\rho^{BC}\right)=0$, and $E_{g}^{\left(3\right)}$ is completely monogamous

(38) $$\begin{array}{c} E_{g}^{\left(3\right)}\left(\rho^{ABC}\right)>E^{\left(2\right)}\left(\rho^{AB}\right) \end{array}$$

is always correct for any $\rho^{ABC}\in S_{g}^{ABC}$. That is, the complete monogamy of $E_{g}^{\left(m\right)}$ refers to it being completely monogamous in the genuine entangled state, and $E_{g}^{\left(m\right)}$ is strictly decreasing under discarding of the subsystem, which is different from that of the complete entanglement measure. Equivalently, if $E_{g}^{\left(3\right)}$ is a continuous complete GMEM, then $E_{g}^{\left(3\right)}$ is tightly complete monogamous if and only if there exists 0<α<∞ such that

(39) $$\begin{array}{c} E_{g}^{\alpha }\left(\rho^{ABC}\right)⩾E^{\alpha }\left(\rho^{AB}\right)+E^{\alpha }\left(\rho^{AB|C}\right) \end{array}$$

holds for all $\rho^{ABC}\in S_{g}^{ABC}$ with fixed $\dim H^{ABC}=d<\infty$, here we omitted the superscript ${}^{\left(3\right)}$ of $E^{\left(3\right)}$ for brevity.

By Definition 4, $E_{g-1234\cdots m(2)}^{F}$ is tightly complete monogamous since for $E_{g-1234\cdots m(2)}^{F}$ the genuine disentangling condition (36) always holds. $C_{gme}$ is not tightly complete monogamous since it violates the genuine disentangling condition. In addition, the tightly complete monogamy of $E_{g}^{\left(m\right)}$ is closely related to that of $E^{\left(m\right)}$ whenever $E_{g}^{\left(m\right)}$ is derived from $E^{\left(m\right)}$ as in Equations (24) or (25).

**Proposition** **4.** *Let $E^{\left(m\right)}$ be a complete multipartite entanglement measure. If $E^{\left(m\right)}$ is tightly complete monogamous, then the genuine multipartite entanglement measure $E_{g}^{\left(m\right)}$, induced by $E^{\left(m\right)}$ as in Equations (24) or (25), is tightly complete monogamous.*

Together with Proposition 4 in Ref. [22], $R_{g-\alpha }^{\left(m\right)}$, $N_{g-F}^{\left(m\right)}$ and $N_{g}^{\left(m\right)}$ are completely monogamous but not tightly complete monogamous.

## 5. Conclusions and Discussion

We have proposed a framework of unified/complete genuine multipartite entanglement measure, from which we established the scenario of complete monogamy and tightly complete monogamy of genuine multipartite entanglement measure. The spirit here is consistent with that of a unified/complete multipartite entanglement measure in Ref. [22]. We also find a simple way of deriving a unified/complete genuine multipartite entanglement measure from the unified/complete multipartite entanglement measure. Under such a framework, the multipartite entanglement becomes more clear, and, in addition, we can judge whether a given genuine entanglement measure is good or not. Compared with other multipartite entanglement measure, the unified genuine entanglement measure is automatically completely monogamous. That is, genuine entanglement displays the monogamy of entanglement more evidently than other measures. These results support that entanglement is monogamous, as we expected. We thus suggest that monogamy should be a necessary requirement for a genuine entanglement measure.

## Data Availability

Not applicable.

## References

[1] Nielsen M.A., Chuang I. (2000). Quantum Computatation and Quantum Information.

[2] Bennett C.H., DiVincenzo D.P., Smolin J.A., Wootters W.K. (1996). Mixed-state entanglement and quantum error correction. Phys. Rev. A.

[3] Horodecki R., Horodecki P., Horodecki M., Horodecki K. (2009). Quantum entanglement. Rev. Mod. Phys..

[4] Gühne O., Tóth G. (2009). Entanglement detection. Phys. Rep..

[5] Burkhart L.D., Teoh J.D., Zhang Y., Axline C.J., Frunzio L., Devoret M.H., Jiang L., Girvin S.M., Schoelkopf R.J. (2021). Error-Detected State Transfer and Entanglement in a Superconducting Quantum Network. PRX Quantum.

[6] Yu X.-D., Imai S., Gühne O. (2021). Optimal Entanglement Certification from Moments of the Partial Transpose. Phys. Rev. Lett..

[7] Luo M.X. (2021). New Genuinely Multipartite Entanglement. Adv. Quantum Technol..

[8] Schmid D., Fraser T.C., Kunjwal R., Sainz A.B., Wolfe E., Spekkens R.W. (2020). Understanding the interplay of entanglement and nonlocality: Motivating and developing a new branch of entanglement theory. arXiv.

[9] Navascués M., Wolfe E., Rosset D., Pozas-Kerstjens A. (2020). Genuine Network Multipartite Entanglement. arXiv.

[10] Coffman V., Kundu J., Wootters W.K. (2000). Distributed entanglement. Phys. Rev. A.

[11] Ma Z.H., Chen Z.H., Chen J.L., Spengler C., Gabriel A., Huber M. (2011). Measure of genuine multipartite entanglement with computable lower bounds. Phys. Rev. A.

[12] Rafsanjani S.M.H., Huber M., Broadbent C.J., Eberly J.H. (2012). Genuinely multipartite concurrence of *N*-qubit *X* matrices. Phys. Rev. A.

[13] Sen(De) A., Sen U. (2010). Channel capacities versus entanglement measures in multiparty quantum states. Phys. Rev. A.

[14] Sadhukhan D., Roy S.S., Pal A.K., Rakshit D., Sen(De) A., Sen U. (2017). Multipartite entanglement accumulation in quantum states: Localizable generalized geometric measure. Phys. Rev. A.

[15] Emary C., Beenakker C.W.J. (2004). Relation between entanglement measures and Bell inequalities for three qubits. Phys. Rev. A.

[16] Contreras-Tejada P., Palazuelos C., de Vicente J.I. (2019). Resource theory of entanglement with a unique multipartite maximally entangled state. Phys. Rev. Lett..

[17] Das S., Bäuml S., Winczewski M., Horodecki K. (2019). Universal limitations on quantum key distribution over a network. arXiv.

[18] Xie S., Eberly J.H. (2021). Triangle Measure of Tripartite Entanglement. Phys. Rev. Lett..

[19] Guo Y., Jia Y.-P., Li X.-P., Huang L.-Z. (2021). Genuine multipartite entanglement measure. arXiv.

[20] Bennett C.H., Bernstein H.J., Popescu S., Schumacher B. (1996). Concentrating partial entanglement by local operations. Phys. Rev. A.

[21] Eltschka C., Siewert J. (2018). Distribution of entanglement and correlations in all finite dimensions. Quantum.

[22] Guo Y., Zhang L. (2020). Multipartite entanglement measure and complete monogamy relation. Phys. Rev. A.

[23] Gour G., Guo Y. (2018). Monogamy of entanglement without inequalities. Quantum.

[24] Bai Y.-K., Xu Y.-F., Wang Z.-D. (2014). General monogamy relation for the entanglement of formation in multiqubit systems. Phys. Rev. Lett..

[25] Streltsov A., Adesso G., Piani M., Bruß D. (2012). Are general quantum correlations monogamous?. Phys. Rev. Lett..

[26] Koashi M., Winter A. (2004). Monogamy of quantum entanglement and other correlations. Phys. Rev. A.

[27] Osborne T.J., Verstraete F. (2006). General monogamy inequality for bipartite qubit entanglement. Phys. Rev. Lett..

[28] Deng X., Xiang Y., Tian C., Adesso G., He Q., Gong Q., Su X., Xie C., Peng K. (2017). Demonstration of monogamy relations for Einstein-Podolsky-Rosen steering in Gaussian cluster state. Phys. Rev. Lett..

[29] Camalet S. (2017). Monogamy inequality for any local quantum resource and entanglement. Phys. Rev. Lett..

[30] Karczewski M., Kaszlikowski D., Kurzyński P. (2018). Monogamy of particle statistics in tripartite systems simulating Bosons and Fermions. Phys. Rev. Lett..

[31] Lancien C., DiMartino S., Huber M., Piani M., Adesso G., Winter A. (2016). Should entanglement measures be monogamous or faithful?. Phys. Rev. Lett..

[32] Ou Y.-C., Fan H. (2007). Monogamy inequality in terms of negativity for three-qubit states. Phys. Rev. A.

[33] Cheng S., Hall M.J.W. (2017). Anisotropic Invariance and the Distribution of Quantum Correlations. Phys. Rev. Lett..

[34] Allen G.W., Meyer D.A. (2017). Polynomial Monogamy Relations for Entanglement Negativity. Phys. Rev. Lett..

[35] He H., Vidal G. (2015). Disentangling theorem and monogamy for entanglement negativity. Phys. Rev. A.

[36] Guo Y., Gour G. (2019). Monogamy of the entanglement of formation. Phys. Rev. A.

[37] Guo Y. (2019). Strict entanglement monotonicity under local operations and classical communication. Phys. Rev. A.

[38] Regula B., Osterloh A., Adesso G. (2016). Strong monogamy inequalities for four qubits. Phys. Rev. A.

[39] Eltschka C., Siewert J. (2015). Monogamy Equalities for Qubit Entanglement from Lorentz Invariance. Phys. Rev. Lett..

[40] Eltschka C., Huber F., Gühne O., Siewert J. (2018). Exponentially many entanglement and correlation constraints for multipartite quantum states. Phys. Rev. A.

[41] Guo Y., Huang L., Zhang Y. (2021). Monogamy of quantum discord. Quantum Sci. Technol..

[42] Hong Y., Gao T., Yan F. (2012). Measure of multipartite entanglement with computable lower bounds. Phys. Rev. A.

[43] Hiesmayr B.C., Huber M. (2008). Multipartite entanglement measure for all discrete systems. Phys. Rev. A.

[44] Guo Y., Zhang L., Yuan H. (2020). Entanglement measures induced by fidelity-based distances. Quant. Inf. Process..

[45] Jozsa R. (1994). Fidelity for mixed quantum states. J. Mod. Opt..

[46] Uhlmann A. (1976). The ‘transition probability’ in the state space of a*-algebra. Rep. Math. Phys..

[47] Zhang L., Chen L., Bu K. (2015). Fidelity between one bipartite quantum state and another undergoing local unitary dynamics. Quant. Inf. Process..

[48] Fawzi O., Renner R. (2015). Quantum conditional mutual information and approximate Markov chains. Commun. Math. Phys..

[49] Luo S., Zhang Q. (2004). Informational distance on quantum state space. Phys. Rev. A.

[50] Ma Z., Zhang F.L., Chen J.L. (2008). Geometric interpretation for the a fidelity and its relation with the Bures fidelity. Phys. Rev. A.

[51] Raggio G.A. (1984). Generalized Transition Probabilities and Applications Quantum Probability and Applications to the Quantum Theory of Irreversible Processes.

[52] Zhu X.N., Fei S.M. (2014). Entanglement monogamy relations of qubit systems. Phys. Rev. A.

[53] Luo Y., Tian T., Shao L.H., Li Y. (2016). General monogamy of Tsallis *q*-entropy entanglement in multiqubit systems. Phys. Rev. A.

[54] Kumar A. (2017). Multiparty quantum mutual information: An alternative definition. Phys. Rev. A.

